# Lingual microbiota profiles of patients with geographic tongue

**DOI:** 10.1080/20002297.2017.1355206

**Published:** 2017-07-27

**Authors:** Amal Dafar, Maria Bankvall, Hülya Çevik-Aras, Mats Jontell, Fei Sjöberg

**Affiliations:** ^a^ Department of Oral Medicine and Pathology, Institute of Odontology, Gothenburg, Sweden; ^b^ Department of Infectious Diseases, Institute of Biomedicine and Department of Oncology, Institute of Clinical Sciences; The Sahlgrenska Academy, University of Gothenburg, Gothenburg, Sweden

**Keywords:** Benign migratory glossitis, tongue lesions, oral mucosal lesions, oral diseases, next-generation sequencing

## Abstract

Geographic tongue (GT) is an oral mucosal lesion that affects the tongue. The association between GT and the bacterial colonization profiles of the tongue is not clear. Lingual swabs were collected from lesion sites and healthy sites of 35 patients with GT (19 males and 16 females; M_age_ = 54.3 ± 16.1 years) and 22 controls (12 males and 10 females; M_age_ = 56.3 ± 15.8 years). Bacterial DNA was extracted and sequenced by next-generation sequencing. At the phylum level, Fusobacteria were significantly less abundant, while Spirochaetes were significantly more abundant in GT patients compared to controls. At the operational taxonomic units level, multivariate analysis revealed distinct clusters for the three groups based on the lingual microbiota composition. Acinetobacter and Delftia were significantly associated with GT lesion and healthy sites. However, Microbacterium, Leptospira, Methylotenera, and Lactococcus were significantly associated with GT lesion sites. Additionally, Mogibacterium and Simonsiella were significantly associated with GT healthy sites and controls. The changes in the lingual microbiota profiles of patients with GT imply a shift in the lingual bacterial ecology. However, it remains unknown if this shift is a consequence of the lesions or of factors associated with the initiation and progression of the disease.

## Introduction

Geographic tongue (GT) is a common oral mucosal lesion that usually affects the dorsal and lateral surfaces of the tongue. The prevalence of GT ranges from 0.5% to 12.7% in different populations [1,2]. GT is characterized by erythematous areas, representing papillary atrophy, surrounded by a whitish peripheral zone. A prominent feature of GT is the migration of the lesions to create a map-like pattern that constantly changes [3]. In general, GT is asymptomatic, although in some cases symptoms, such as soreness, sensitivity, and burning sensations, are elicited by the intake of acidic drinks and spicy foods [4–7]. Several factors have been identified as being associated with GT, including the presence of systemic diseases [4,8–10] and stress [7,11,12]. Microbial factors, mainly the presence of fungi, have also been associated with GT. *Candida* has been reported as being commonly found in GT lesions [7,13,14].

Next-generation sequencing (NGS) has revolutionized the study of microbial diversity [15]. Oral microbiome analyses in health and disease are currently being used to gain a better understanding of oral microbial diseases [16]. Recently, the roles of the oral microbiota in some oral mucosal lesions, such as recurrent aphthous ulceration [17,18] and oral lichen planus [19], have been reported.

In patients with GT, the normal tongue anatomy, characterized by the presence of papillae, is changed due to papillary atrophy, which is evident clinically. This change in the tongue structure may lead to dysbiosis of the lingual microbiota, which in turn might trigger the inflammation. It was previously found that GT is an inflammatory condition, in which the levels of salivary interleukin-8 (IL-8), a pro-inflammatory mediator and a chemotactic factor, were found to be approximately twofold higher in patients with GT than they were in controls [20]. Another suggestion is that GT is caused by dysbiosis of the lingual microbiota. Thus, a vicious circle may be involved in GT pathogenesis.

The aim of this study was to define the lingual microbiota profiles of patients with GT, at both the lesion sites and the surrounding healthy sites. Moreover, both sites were compared to the lingual microbial profiles of healthy control individuals.

## Methodology

### Study population

In this cross-sectional case-control study, a total of 57 subjects (35 patients with GT and 22 controls) participated. Patients were examined at the Clinic of Oral Medicine, Public Dental Health, Gothenburg, Sweden, or at a private dental clinic in the nearby town of Borås, Sweden. Inclusion criteria were the presence of GT lesions on the surface of the tongue. GT diagnosis was based on the clinical features of the lesions, as well as the history of migratory pattern provided by the patients. Exclusion criteria included the presence of other oral mucosal lesions, such as aphthous ulcers or oral lichen planus, use of antibiotics, or the use of an antibacterial mouth rinse and tobacco. An evaluation of the medical history revealed that 60% of the patients were systemically healthy. The remainder showed high variability in their medical backgrounds. Among these patients, the presence of the following conditions was noted: cardiovascular disease (i.e. hypertension or heart failure; *n* = 5); psoriasis (*n* = 4); fibromyalgia (*n* = 2); rheumatoid arthritis (*n* = 2); asthma (*n* = 2); diabetes (*n* = 2); inflammatory bowel disease (*n* = 2); and vaginal lichen planus (*n* = 1).

The age- and sex-matched controls included staff members of the two clinics, laboratory personnel, and relatives or friends of the patients. The inclusion criteria for the controls were a clinically healthy oral mucosa as well as no use of tobacco. Exclusion criteria included use of antibiotics or an antibacterial mouth rinse. Various illnesses were noted for 54.5% of the controls, including cardiovascular disease (*n* = 6), allergy (*n* = 3), rheumatoid disease (*n* = 1), vaginal lichen planus (*n* = 1), and benign prostatic hyperplasia (*n* = 1).

The Central Ethical Review Board in Gothenburg, Sweden, approved the study (Dnr. 032–12). Written informed consent was obtained from all the participants before they were interviewed.

### Sample collection

Sterile swabs (Isohelix DNA Buccal Swab; Isohelix/Cell Projects Ltd., Harrietsham, Kent, UK) were used to collect bacterial samples according to the manufacturer’s instructions, with the exception of the site of sampling. Samples were collected from the tongue mucosa instead of the buccal mucosa, since the aim was to identify the bacteria on the tongue surface where the GT lesions were located.

In total, 91 tongue samples were collected from 35 patients with GT and from 22 controls. The samples were assigned to four groups: (1) GT-lesion sites (*n* = 34) collected from the erythematous area of the lesion; (2) GT-healthy sites (*n* = 35) collected from the adjacent healthy tongue mucosa; (3) paired samples, that is, lesion and healthy sites of the same individual (*n* = 33); and (4) controls (*n* = 22). One patient gave only one lesion sample, since GT was covering almost the whole surface of the tongue. Thus, the total number of GT-lesion samples was 34. One patient donated only one healthy sample, as we could not detect any GT lesions at the time of sampling. In addition, one patient gave both a lesion sample and a healthy sample, but the lesion sample was excluded, as it did not pass the DNA quality criteria. Thus, the total number of GT-healthy samples was 35 (Supplementary Table S1). The participants were requested not to brush their teeth or consume any food or beverages for at least 1 h prior to the sampling of their tongue mucosa. All the samples were collected by the same dentist.

### DNA extraction

DNA extraction was performed using the QIAamp DNA Stool Mini Kit (Qiagen, Hilden, Germany) according to the manufacturer’s instructions, with some modifications. Briefly, lysis buffer (ASL; 1.3 mL) was added to the swabs, which were vortexed for 1 min, incubated at 37°C for 10 min, and then heated at 95°C for 5 min, followed by vortexing for an additional 15 s. To maximize the DNA yield, five glass beads (3.0 mm diameter) and 0.25 g zirconia beads (0.1 mm diameter) were added to the ASL buffer, and the suspension was homogenized twice at 6 m/s for 40 s (FastPrep Cell disrupter; Thermo Savant, Holbrook, NY). Thereafter, the samples were reheated at 95°C for 5 min and vortexed for 15 s. Soon after, the samples were placed on a shaker at 1,200 rpm (Vibrax Shaker; IKA, Staufen, Germany) for 30 min at 5°C, and then centrifuged for 3 min. Thereafter, the manufacturer’s instructions were followed. The obtained DNA was quantified using a spectrophotometer (NanoDrop Technologies, Wilmington, DE).

### Polymerase chain reaction and sequencing

The DNA samples were sent to GATC Biotech (Konstanz, Germany) to perform the NGS analysis. Polymerase chain reaction (PCR) amplification of the V3–V5 region of the bacterial 16S rRNA genes was performed using forward primer 357F (5′-CCTACGGG-AGGCAGCAG-3′) and reverse primer 926R (5′-CCGTCAATTCMTTTRAGT-3′). Sequencing was performed using the Illumina HiSeq sequencing system, according to standard operating procedures, with read length 300 bases in paired-end sequencing mode.

### Sequence analysis

Read pairs were merged by overlapping the forward and reverse sequence reads using FLASh [21], and the reads with a quality score of <25 were removed. Clustering was carried out based on sequence similarity, with a minimum pairwise identity of 97% using a cd-hit program [22]. Singletons and possible chimeric sequences were removed with UCHIME [23] to minimize the sequencing errors. Thus, operational taxonomic unit (OTU) assignment was performed for non-chimeric and unique clusters using a BLAST analysis [24] for non-redundant 16S rRNA reference sequences, which were obtained from the Ribosomal Database Project [25]. Taxonomic assignment was based on NCBI Taxonomy [26].

### Statistical analyses

The statistical analyses were performed using binary data, which explains the presence or absence of taxa at the OTU level.

Univariate analyses of the relative abundance, the overall richness, and the Shannon and Simpson diversity indexes at the OTU level were performed using one-way analysis of variance (alternatively, the Kruskal–Wallis test) to compare the GT-lesion, GT-healthy, and control groups using Prism 6 software (GraphPad, Inc., La Jolla, CA). Paired *t*-tests (alternatively, the Wilcoxon signed-rank test) were used to compare paired samples (i.e. GT-lesion and GT-healthy) using SAS v9.1 (SAS Institute, Inc., Cary, NC). Statistical corrections for multiple comparisons were performed using Bonferroni or Tukey’s methods.

Multivariate analyses (SIMCA P+ v14.1; Umetrics AB, Umeå, Sweden) were used to obtain the overall pattern of the lingual microbiota in the three groups. This was achieved using a principal component analysis (PCA) and orthogonal partial least square analysis (OPLS). PCA is an unsupervised analysis that depicts the inherent structure of multivariate data and major correlations between observations and variables. In contrast, OPLS is a supervised analysis, and it represents a regression variety of the PCA that relates the *x* data set (the presence or absence of each OTU) to a *y* variable, in this case the diagnosis of GT (i.e. the GT-lesion and GT-healthy groups or controls). OTUs that did not contribute in a substantial way to the model were not shown in the corresponding column plots. Subsequently, a univariate analysis was performed using Fisher’s exact tests (or paired *t*-tests) to confirm unequal distributions between the diagnostic groups of those OTUs that showed the strongest contributions in the respective OPLS models. For all the tests, the level of statistical significance was set at *p <* 0.05.

## Results

### Similar numbers of sequence reads for the lingual microbiota of patients with GT and controls

In total, 91 samples were collected from patients with GT and from controls, as shown in Table 1. The total number of sequences for all the samples was 44,109,902, with an average of 484,724 reads/sample (Supplementary Table S1). An almost equal distribution of reads, without statistically significant differences, was observed between the patients with GT and the controls. This result implies successful extraction of the genetic material across all the samples.

**Table 1.** Characteristics and sampling of patients with geographic tongue (GT) and healthy age- and sex-matched controls

	Patients with GT (*n* = 35)	Control (*n* = 22)
Males (%)	*n* = 19 (54.3%)	*n* = 12 (54.5%)
Females (%)	n = 16 (45.7%)	*n* = 10 (45.5%)
Age (mean ± *SD*)	54.3 ± 16.1	56.3 ± 15.8
Sampling	*n* = 69 (*n* = 34 GT-lesion) (*n* = 35GT-healthy)	*n* = 22

*SD*, standard deviation.

### Distribution of bacterial phyla in the lingual microbiota among patients with GT and controls

**Figure 1.**
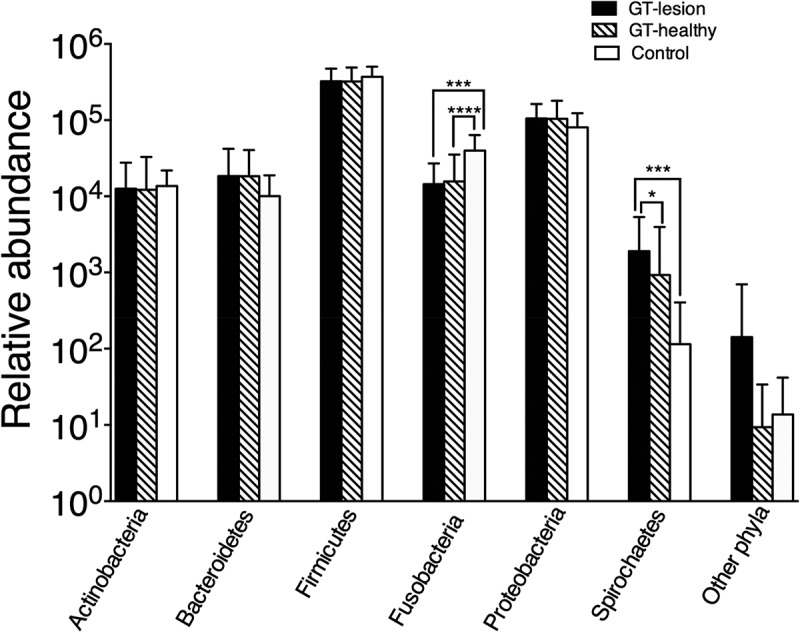
Relative abundance of the bacterial phyla among patients with geographic tongue (GT) and controls. Values shown are means ± standard deviation (*SD*). Comparisons between the groups were analyzed by the Kruskal–Wallis test; *p < 0.05; ***p < 0.001; ****p < 0.0001. The group ‘other phyla’ comprises the five phyla listed in Table 2.

Figure 1 shows the relative abundance of the bacterial phyla for each group. Across all the samples, the following six most abundant bacterial phyla were identified: Firmicutes (69.0%), Proteobacteria (20.3%), Fusobacteria (4.3%), Bacteroidetes (3.3%), Actinobacteria (2.6%), and Spirochaetes (0.2%). Fusobacteria were significantly less abundant among patients with GT, in both the lesion and healthy sites, compared to the controls (*p <* 0.001). In contrast, Spirochaetes were more abundant among GT-lesion sites than in GT-healthy sites (*p <* 0.05) or in the controls (*p* < 0.001). ‘Other phyla’ seen in Figure 1 represent the remaining 0.3% containing five phyla (Table 2) and showed no significant differences between the groups.

**Table 2.** Other phyla^a^ identified among patients with GT and controls

	Number of reads			
Phylum	Total	GT-lesion	GT-healthy	Control
Deinococcus-Thermus	3,374	3,286	80	8
Tenericutes	1,198	793	199	206
Acidobacteria	937	832	21	84
Nitrospirae	75	57	18	0
Fibrobacteres	4	0	0	4

^a^Other phyla that were demonstrated in Figure 1.

### Distribution of bacterial taxa in the lingual microbiota among patients with GT and controls

The relative abundance for the taxa that represent >1.0% of the total reads across all the samples is demonstrated in Table 3. *Streptococcus* represents the most common taxon (48.4%). Most of the identified taxa (7/13) belong to the phylum Firmicutes. In general, controls had higher relative abundance (median) of the commonly found commensals than patients with GT including both GT-lesion and GT-healthy.

**Table 3.** Relative abundance of the bacterial taxa that represent >1% of the total reads

		Median (range) % of the relative abundance		
Taxon (phylum)	Relative abundance, %^a^	Control	GT-lesion	GT-healthy
*Streptococcus* (Firmicutes)	48.4	4.0 (1.5–12.7)	2.5 (0.2–5.8)	2.7 (0.6–7.1)
*Haemophilus* (Proteobacteria)	11.1	3.5 (0.6–10.1)	2.3 (<0.1–9.1)	2.1 (0.3–10.7)
*Veillonella* (Firmicutes)	10.6	3.6 (1.1–12.7)	1.9 (<0.1–11.2)	2.8 (<0.1–6.0)
*Neisseria* (Proteobacteria)	6.6	2.5 (<0.1–11.4)	1.0 (<0.1–27.7)	1.3 (<0.1–23.3)
*Fusobacterium* (Fusobacteria)	4.0	4.3 (<0.1–9.9)	1.9 (<0.1–10.2)	1.1 (<0.1–18.4)
*Granulicatella* (Firmicutes)	2.0	4.1 (0.4–8.5)	2.4 (0.1–12.0)	2.6 (<0.1–8.5)
*Gemella* (Firmicutes)	1.9	3.9 (0.4–13.3)	1.5 (0.0–9.1)	2.3 (0.1–9.0)
*Staphylococcus* (Firmicutes)	1.8	0.3 (0.0–45.8)	0.2 (0.0–36.1)	0.04 (0.0–53.6)
*Vagococcus* (Firmicutes)	1.6	4.6 (0.3–9.6)	2.2 (0.1–9.8)	2.3 (<0.1–9.9)
*Prevotella* (Bacteroidetes)	1.5	3.1 (0.3–16.8)	1.5 (0.0–32.1)	1.2 (0.0–14.5)
*Flavobacterium* (Bacteroidetes)	1.5	0.5 (0.0–61.2)	0.4 (0.0–22.8)	0.1 (0.0–37.5)
*Actinomyces* (Actinobacteria)	1.4	4.8 (0.3–10.6)	1.4 (<0.1–17.2)	1.9 (<0.1–14.2)
*Enterococcus* (Firmicutes)	1.2	3.0 (0.2–24.2)	1.9 (0.1–18.8)	1.8 (0.0–22.8)

^a^Relative abundance for each taxon was calculated as number of reads for the taxon divided with the total number of all the reads across all the samples.

### Richness and diversity of the lingual microbiota in patients with GT and controls

**Figure 2.**
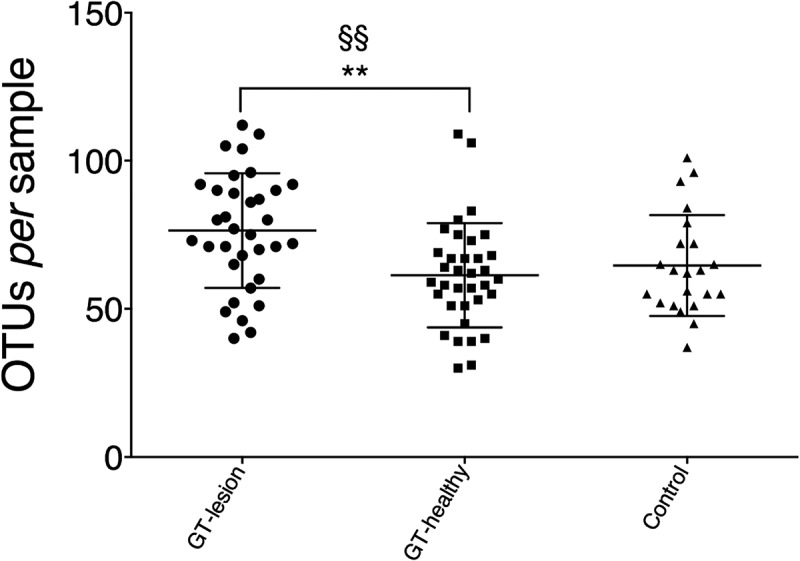
Richness of the lingual microbiota in patients with GT and controls. Bacterial DNA was extracted and the 16S rRNA genes were sequenced and assigned to operational taxonomic units (OTUs) with a minimum pairwise identity of 97.0%. Each symbol represents one individual. Values shown are means ± *SD*. Comparisons between the groups were analyzed by one-way analysis of variance (ANOVA), **p < 0.01, and a paired *t*-test ^§§^*p* < 0.01.

To assess microbiotal richness, the numbers of OTUs per patient were calculated (Figure 2). The GT-lesion group showed a higher mean number of OTUs compared to the GT-healthy group (75.8 vs. 61.5; *p* < 0.01). However, no differences were found in a comparison of the two GT groups with the controls (mean number of OTUs for the controls was 64.5). Comparing the paired samples using a paired *t*-test yielded the same finding (*p* < 0.01) for the GT-lesion and GT-healthy groups. The finding that fewer OTUs were identified in the GT-healthy group than in the GT-lesion group may reflect the lower total numbers of bacteria in the GT-healthy sites.

**Figure 3.**
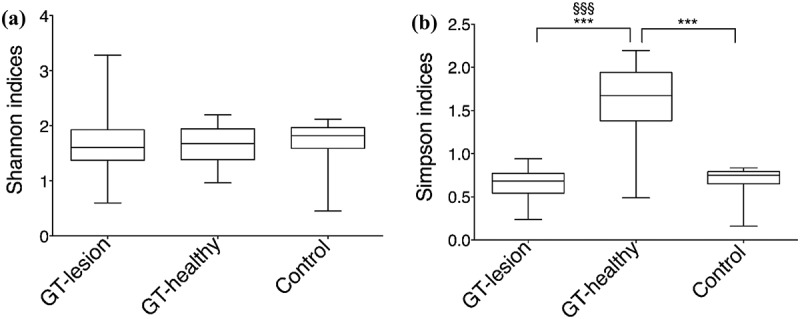
Diversity indexes at the OTU level. (a) Shannon’s index values; (b) Simpson’s index values. Comparisons between the groups were analyzed by one-way ANOVA, ***p < 0.001 (comparisons between all groups), and Wilcoxon’s signed-rank test, ^§§§^*p* < 0.001 (comparisons between the paired samples). The 25th and 75th percentiles are shown in the box plot. The median is indicated by the horizontal solid lines. The bars indicate the minimum to maximum values.

The diversity indexes, Shannon and Simpson, were compared at the OTU level, and it was found that the Shannon diversity index showed no differences between the three groups (Figure 3a). In contrast, the Simpson index showed that the bacterial abundance at GT-healthy sites was significantly higher than at GT-lesion sites and in the controls (medians = 1.67 vs. 0.68 vs. 0.75; *p* < 0.001). Moreover, comparisons of paired samples showed a significantly higher Simpson index in the GT-healthy group than in the GT-lesion group (medians = 1.67 vs. 0.68; *p* < 0.001) levels (Figure 3b).

### Differences in the compositions of the lingual microbiota of patients with GT and controls

**Figure 4.**
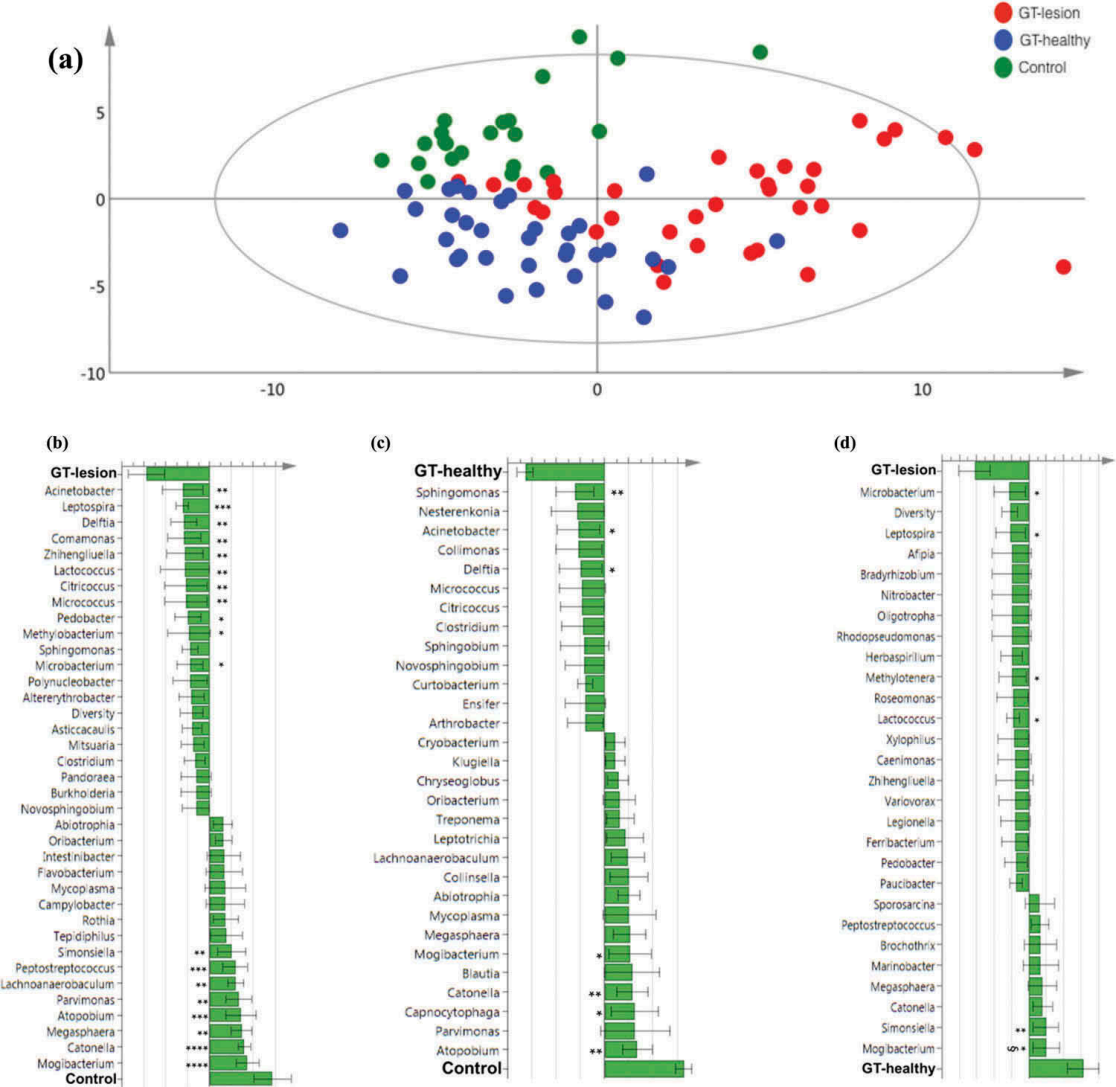
Differences in the lingual microbiota compositions of patients with GT and controls. The presence or absence of 403 identified OTUs was checked in each individual of the three groups. (a) Principal component analysis. Each symbol represents one individual. The 2D illustration shows that subjects that belong to the same group tend to cluster together, whereas those that belong to different groups remain separated from one another. (b–d) Orthogonal partial least square analysis loading plots. Shown are those OTUs that contribute most to distinguishing between the GT-lesion group and the control group (b), the GT-healthy group and the control group (c), and the GT-lesion group and the GT-healthy group (d). OTUs that show the strongest contribution to the respective models were tested for differences in prevalence using Fisher’s exact tests (or paired *t*-tests for paired samples). *p < 0.05; **p < 0.01; ***p < 0.001; and ^§^*p* < 0.05 for paired *t*-test.

In the data set, a total of 403 OTUs were identified. The presence or absence of each of the 403 OTUs was identified in each individual of the GT-lesion, GT-healthy, and control groups. Differences in microbiota composition were analyzed using a multivariate model (PCA; Figure 4a) and showed that subjects who belonged to the same group tended to cluster together, in contrast to the subjects from different groups who tended to separate from one another.

### Comparison of the GT-lesion and control

The OTUs that contributed most to discriminating the GT-lesion group from the control group are illustrated in Figure 4b. Bars pointing left represent microbiota that are more prevalent in the GT-lesion group, while bars pointing right represent microbiota that are more prevalent in the control group. The taller the bar, the stronger the contribution is to the model. In the GT-lesion group (upper part of the figure), the significantly more prevalent phyla (with corresponding genera) were: Proteobacteria (*Acinetobacter*, *Delftia, Comamonas*, and *Methylobacterium*); Actinobacteria (*Zhihengliuella*, *Citricoccus, Micrococcus*, and *Microbacterium*); Spiro-chaetes (*Leptospira*); Firmicutes (*Lactococcus*); and Bacteroidetes (*Pedobacter*). In contrast, the following phyla (with corresponding genera) were found to be significantly more frequent among the controls (lower part of the figure): Firmicutes (*Mogibacterium*, *Catonella*, *Megasphaera*, *Parvimonas, Lachnoanaerobaculum*, and *Peptostreptococcus*); Actinobacteria (*Atopobium*); and Proteobacteria (*Simonsiella*).

### Comparison of the GT-healthy and control

In a comparison of the GT-healthy and control groups (Figure 4c), it was observed that three genera, belonging to the phylum Proteobacteria, were present more often among the GT-healthy group: *Sphingomonas, Acinetobacter*, and *Delftia*. In contrast, among the control group, the following phyla (genera) were significantly more frequent: Firmicutes (*Mogibacterium* and *Catonella*), Actinobacteria (*Atopobium*), and Bacteroidetes (*Capnocytophaga*).

### Comparison of the GT-lesion and GT-healthy (including paired t-test)

Furthermore, when the microbiota of the GT-lesion and GT-healthy groups were compared (Figure 4d), four (genera) belonging to four different phyla were found to be significantly associated with the presence of GT lesions: Actinobacteria (*Microbacterium*), Spirochaetes (*Leptospira*), Proteobacteria (*Methylo-tenera*), and Firmicutes (*Lactococcus*). In contrast, Fimicutes (*Mogibacterium*) and Proteobacteria (*Simonsiella*) were found at significantly higher frequencies in the GT-healthy group. However, a paired *t*-test showed that *Mogibacterium* was the only taxon that showed a significantly different frequency between the two groups (*p <* 0.05).

## Discussion

To the authors’ knowledge, this is the first study to show an association between GT and dysbiosis of the lingual microbiota. Dysbiosis is defined as changes to the composition of the commensal microbiota that involve three mechanisms: (1) loss of beneficial bacteria, (2) expansion of the populations of pathogenic bacteria, and (3) changes in the diversity of the microbiota [27]. This study focused on the importance of microbiota composition and diversity rather than the loss of beneficial bacteria or the expansion of populations of potentially pathogenic bacteria, since it was hypothesized that no specific pathogen is associated with GT but rather GT represents a compositional change of the overall microbial community on the tongue. Using NGS, it was possible to identify the predominant taxa on the tongues of patients with GT. Collectively, the identified taxa showed different microbiota compositions at the lesion sites and healthy sites in the patients with GT compared to healthy age- and sex-matched controls.

The normal tongue structure, which is characterized by the presence of papillae, creates a niche in which mechanical retention of bacteria is facilitated by the papillae, reducing the risk of bacteria being flushed away by the saliva [28]. However, the most striking feature of GT is the loss of the filiform papillae [3]. Therefore, it is plausible that the niches provided by the papillae are lost in GT. Consequently, the normal ecology of the tongue is changed and, as a direct consequence, the immune system is induced to initiate inflammation [29]. It has been shown that the changes in microbial diversity underlie the dramatic and rapid increases in chronic inflammatory and autoimmune disorders seen in high-income countries [30–32]. Increasing evidence suggests that caries and periodontal diseases represent dysbiotic states of the oral microbiome [33].

A major finding in the present study is that lesion sites of GT show a significantly greater richness (i.e. more bacterial taxa were identified [as OTUs]) in the lesion sites than in the healthy sites of the GT. However, the greater richness of the bacteria in the lesion sites does not imply a higher bacterial density, since the number of sequence reads was equally distributed across all the samples. The greater richness of the bacteria in the lesions may be explained by a significantly increased prevalence of the phylum Spirochaetes. Another contributing factor is the higher number of reads in the group of other phyla, although this was not statistically significant. The study also demonstrated that the richness of healthy sites of GT is slightly lower than for the controls, albeit not significantly so. The explanation for this is that the healthy sites of GT display a normal tongue structure, which is suitable for ‘commensal’ bacteria to take up residence. However, the nearby lesion sites of GT lack this normal structure with papillae, which means that the microbiota is altered and it becomes difficult to re-establish a normal ecology.

The Shannon and Simpson indexes are measures of diversity. These indexes are based on two components: richness, which is the number of species per sample; and evenness, which is the relative abundance of species. The results show that Shannon’s index revealed no differences between the groups. Since Shannon’s index is an information statistical index that assumes that all species are represented in a sample, with the result that evenness becomes less important and richness takes on greater importance (represented here as an almost equal number of sequence reads across all the groups). In contrast, Simpson’s index is a dominance index that assigns greater weight to common or dominant species. In this case, rare species with only a few representatives will not affect the diversity. Furthermore, a higher Simpson’s index indicates a more even distribution of bacteria. The present results show that Simpson’s index was significantly higher for the healthy sites in GT. The interpretation of this is that the healthy sites of GT have significant taxa that belong to Firmicutes and Proteobacteria, which are the dominant phyla, while at the same time the taxa may be evenly distributed. This situation is unfavorable because the oral microbiota is normally not evenly distributed. In fact, the lower Simpson’s index among the controls represented an uneven distribution, which in turn reflected the normal biological state [27].

In the Human Microbiome Project (HMP), a detailed description of the bacterial community composition of the tongue dorsum has been given [34]. To a great extent, the prevalence of the most commonly found taxa from the present results were in line with the results of the HMP. In the oral cavity, it is well-known that the Firmicutes phylum predominates, since streptococci, which belong to this phylum, constitute the majority of the bacteria residing within the mouth [35]. In fact, the finding of an almost equal distribution of Firmicutes among patients with GT and controls validated the sample collection in the sense that it was possible to show that Firmicutes is still the predominant phylum, even in the presence of a pathological condition such as GT. In contrast, significantly fewer Fusobacteria were found in the patients with GT. Fusobacteria play a role in the induction of human β-defensin-2, which exerts antimicrobial activities for maintaining a healthy mucosal surface [36]. Accordingly, it may be assumed that a reduction in the number of Fusobacteria in GT is associated with inflammation. The finding of higher numbers of Spirochaetes in GT is in line with another study that demonstrated the role of Spirochaetes in oral infections [37]. In contrast to the finding of fewer Fusobacteria and higher numbers of Spirochaetes, several studies have shown that fusospirochetal bacteria are increasing in acute stomatitis and acute ulcerative gingivitis [38,39].

The multivariate analysis results suggested that there are differences in the lingual microbiota compositions between the GT-lesion, GT-healthy, and control groups at the OTU level. It is important to emphasize that the number of significant OTUs belonging to Firmicutes was higher in the control group than in the GT-lesion group (six vs. one). Despite the greater diversity of bacteria at the lesion sites of patients with GT, the reduced diversity of Firmicutes in GT should have an impact on the oral microbiota ecology.

Two genera, *Acinetobacter* and *Delftia* (both belonging to the Proteobacteria phylum), were identified as being associated with GT, regardless of whether the samples were collected from lesions or healthy sites of the GT. In line with these results, the abundance of *Acinetobacter* was significantly higher among patients with recurrent aphthous ulcers [40]. In contrast to these findings, a previous study showed that *Delftia* was associated with healthy root sites rather than root sites with caries [41].

This study also demonstrate that four bacterial OTUs are associated with the lesion sites of GT: *Microbacterium*, *Leptospira*, *Methylotenera*, and *Lactococcus. Microbacterium* was found previously to be associated with refractory periodontitis [42]. To the best of the authors’ knowledge, *Leptospira* has not previously been identified in oral lesions. However, a genus that is closely related to *Leptospira* has been identified in periodontitis [43]. This study showed that *Leptospira* occured in 88.5% of GT lesion sites. It has been demonstrated in an experimental animal model that the human saliva and the oral mucosal barrier provide a natural defence against oral infections by *Leptospira* [44]. Therefore, the breakdown of the mucosal barrier that occurs in GT due to the loss of a normal tongue structure may explain the higher numbers of *Leptospira* in the GT lesions. *Methylotenera* is a newly defined genus [45,46], and data regarding its clinical implications are currently lacking. *Lactococcus* has been demonstrated to play an indirect role in the prevention of caries by competing with cariogenic bacteria [47].

Two genera were identified in the healthy sites of the GT and control groups: *Mogibacterium* and *Simonsiella*. Interestingly, the numbers of *Mogibacterium* were found to be increased in periodontal pockets and root canals after chemo-mechanical preparation of root canals [48], which indicated an association with more healthy structures. Similar to these results, *Simonsiella* was found in 22% of patients with a healthy oral mucosa compared to 8% of patients with erosive lichen planus. The same study showed that 95% of *Simonsiella* colonized the dorsal surface of the tongue [49].

In the present study, the sampling procedure may have had an impact on the number of harvested bacteria, even though all the samples were collected by the same investigator following the manufacturer’s instructions. It is possible that the GT-lesion group already had a greater bacterial load than the GT-healthy group or the control group due to the anatomical changes caused by the disease. Nevertheless, the sequence reads showed an almost equal distribution of reads across all the samples.

The resolution capacity of the heterogeneous 16S rRNA genes varies across bacterial taxa, and occasionally, different members of a family cannot be resolved. The reasons for this are: (1) the fit of the universal PCR primers, as it has been shown that universal primers may not be entirely suitable for the detection of *Bifidobacteria*, belonging to the Actinobacteria phylum, due to their high GC (guanine-cytosine) content [50]; (2) the number of 16S operon copies is not the same in all bacterial species, with a range of 1–15 copies being suggested [51]; and (3) the efficacy of bacterial DNA extraction. DNA can be extracted easily from Gram-negative bacteria, while some Gram-positive bacteria have a very thick and sturdy cell wall that makes DNA extraction more difficult. However, a Qiagen extraction kit was used, which shows greater efficacy than other kits [52]. Moreover, glass bead beating was employed to enhance breaking of the bacterial cell wall [53].

A limitation of this study was the question related to the viability of the collected bacteria, which has ramifications for the DNA-based methods. However, the chances of finding dead bacteria on the tongue surface are quite low due to the washing effect of saliva, the mechanical friction caused by the movement of food, and the continuous turnover of the oral epithelium [54]. Another limitation was the lack of data regarding caries and periodontal status. Differences between patients and controls regarding oral hygiene and periodontal health were not noticed.

A future plan is to conduct a longitudinal study, in which the lingual microbiota is sampled in different phases of the disease (i.e. exacerbations and remissions). This gives an opportunity to assess if the microbial ecology shift preceding or succeeding the appearance of the lesions. It is of interest to perform such a study in children, who may suffer from symptoms related to their tongues but are less likely to have other systemic diseases that may influence the composition of the oral microbiota.

In conclusion, the microbiota associated with GT is complex. Several taxa predominated in both the GT-lesion and GT-healthy groups. It is fundamental to understand the processes that underlie the development and stability of microbial populations in the healthy mouth in order to figure out how these populations are transformed into a dysbiotic state in disease [33]. It remains unknown whether the change in the composition of the tongue microbiota is a direct consequence of GT or is linked to factors that are associated with the initiation and progression of this condition. Despite the daily challenges related to the intake of food and drinks and exposure to other micro-organisms, the make-up of the oral microbiota is relatively stable [55], as evidenced by the abundance of Firmicutes. However, a shift in the bacterial community, due to an increase or decrease in the bacterial composition or diversity, is unfavorable and may be of clinical importance.

## Supplementary Material

Supplementary_Table_1.xlsx
